# High‐Power Hybrid Nanogenerator With Multi‐Degree‐of‐Freedom Mechanical Coupling for Harvesting Water Wave Energy

**DOI:** 10.1002/advs.77154

**Published:** 2026-08-14

**Authors:** Shuai Ding, Hua Zhai, Hao Gong, Xiao Long, Yuanzhuo Tian, Zhanyong Hong, Long Zheng, Xucong Wang, Xiangyu Chen, Zhong Lin Wang

**Affiliations:** ^1^ Anhui Province Key Lab of Aerospace Structural Parts Forming Technology and Equipment Institute of Industry & Equipment Technology Hefei University of Technology Hefei China; ^2^ Beijing Key Laboratory of High‐Entropy Energy Materials and Devices Beijing Institute of Nanoenergy and Nanosystems Chinese Academy of Sciences Beijing China; ^3^ School of Nanoscience and Engineering University of Chinese Academy of Sciences Beijing China; ^4^ College of Polymer Science and Engineering State Key Laboratory of Polymer Materials Engineering Sichuan University Chengdu China

**Keywords:** an electromagnetic generator pump, cable monitoring, hybrid nanogenerator, multi‐degree‐of‐freedom mechanical coupling, water wave energy harvesting

## Abstract

Wave energy is an important renewable energy source, but its low frequency and irregular amplitude make it difficult to harvest effectively, limiting the application of traditional generators. In this work, we report a high‐power hybrid nanogenerator (HP‐HNG) with multi‐degree‐of‐freedom mechanical coupling, which consists of a transmission system and four power generation modules (PGMs), each comprising an electromagnetic generator (EMG) pumping a main triboelectric nanogenerator (M‐TENG), a rotational electromagnetic generator (R‐EMG), and two rotational triboelectric nanogenerators(R‐TENGs). Water waves can be harvested through the heave and pitch of the HP‐HNG, transforming multi‐degree‐of‐freedom energy input into a single output. Part of the EMG as a charge pump, outputting rectified positive and negative charges into the M‐TENG via voltage‐doubling rectification, achieving ultra‐high surface charge density. The HP‐HNG can reach a peak output power of 0.764 W, corresponding to a peak power density of 127.303 W m^−^^3^. With a water wave frequency of 1 Hz, the HP‐HNG can charge a 4.7mF capacitor to 2.5 V in 2 s. The HP‐HNG enables real‐time monitoring of submarine cable temperature and displacement through thermometers and other sensors. This work provides a constructive technical pathway for intelligent monitoring of submarine cables, demonstrating strong potential in distributed sensing applications for the marine Internet of Things.

## Graphical abstract

A high‐power hybrid nanogenerator (HP‐HNG) with multi‐degree‐of‐freedom mechanical coupling is reported, integrating multi‐directional wave energy for output to achieve 1 + 1 > 2 energy harvesting. The HP‐HNG achieves a peak output power of 0.764 W, corresponding to a peak power density of 127.303 W m^−^^3^. At a wave frequency of 1 Hz, the HP‐HNG delivers 111.06 mJ of energy within 5 s under pendulum + heave motion. This work demonstrates the HP‐HNG's capability to simultaneously harvest multi‐degree‐of‐freedom wave energy, providing sustainable power for future submarine cable monitoring and distributed sensing systems in the marine Internet of Things.

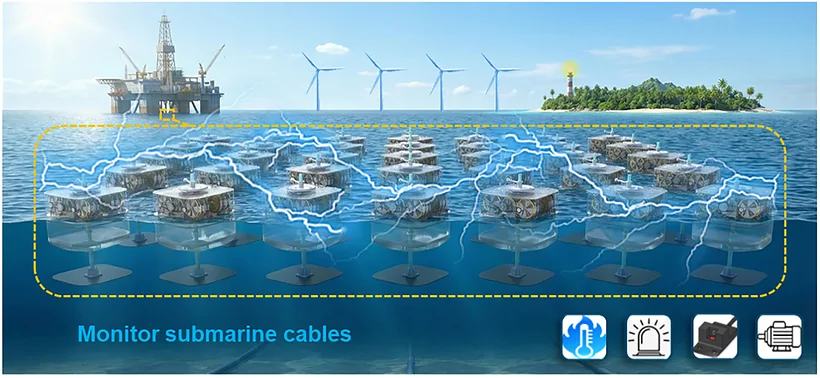

## 1 Introduction

Energy is the material foundation for the survival and development of human society, and the reasonable development and utilization of energy play a greatly positive role in human progress [1, 2, 3]. Based on the advantages of large reserves and wide distribution, marine energy is regarded as one of the important clean energy sources [4, 5, 6, 7]. With the rapid development of science and technology, the development of marine IoT technologies based on renewable energy harvesting and high‐precision sensing provides important technical support for humans to utilize and manage the ocean [8, 9, 10]. The unpredictable weather conditions at sea can easily cause malfunctions in certain devices, such as solar panels, leading to the loss of monitored ocean data during this critical period. At the same time, wave energy becomes more abundant under harsh sea conditions, offering the potential for round‐the‐clock ocean monitoring [11, 12, 13]. The comprehensive ocean monitoring system encompasses multiple aspects, including surface vessels, surface infrastructure, the marine environment, and subsea infrastructure, with the monitoring of submarine cables being particularly crucial. Submarine cables are in a complex marine environment and are easily damaged by external forces, such as ship anchors and marine organisms [14, 15, 16]. Therefore, real‐time monitoring of submarine cable status is essential for promptly detecting potential faults and ensuring the safe and stable operation of power systems [17, 18].

Triboelectric nanogenerators (TENGs), as a new type of energy harvesting technology, are widely applied in self‐powered sensors, micro‐nano energy, and high‐voltage power sources due to their advantages of diverse material selection, low cost, and diverse structural designs [19, 20]. Furthermore, unlike traditional TENGs, which rely on friction at the material surface to generate static charges, the injected confined charges in pumped TENGs can produce an electric field equivalent to that generated by frictional static charges; however, the charge density is theoretically limited only by the breakdown strength of the dielectric material [21]. The charge pumping strategy decouples the charge generation process from the charge output process, allowing the main TENG to adopt a non‐contact design. This significantly reduces mechanical wear while maintaining ultra‐high output performance, thereby markedly improving the device's long‐term operational stability and durability [22, 23]. In contrast to electromagnetic generators (EMGs), TENGs, which are based on the principles of triboelectric charging and electrostatic induction coupling, have displacement current characteristics within the material, allowing them to effectively harvest energy from low‐frequency environments [24, 25]. Integrating TENG with EMG to harvest ocean energy, allowing the hybrid nanogenerator to broaden its frequency bandwidth for energy harvesting and significantly enhance energy harvesting capabilities in confined spaces. The integration of TENG and EMG enables generators to simultaneously leverage the advantages of high voltage and current, overcoming the mismatch between voltage and current when the two technologies operate separately [26, 27]. Although hybrid energy harvesting technologies for ocean energy harvesting have been extensively studied in recent years, various structured hybrid nanogenerators‐such as spherical shell and spring‐assisted structures‐have been designed to harvest wave energy through interconnected networks of hybrid nanogenerators [28, 29, 30]. However, most hybrid nanogenerators can only harvest the kinetic energy of waves in the direction of internal object movement, or the gravitational potential energy of waves in the vertical direction, which makes it nearly impossible to efficiently harvest multi‐degree‐of‐freedom energy from waves simultaneously [31, 32, 33]. Therefore, developing a multi‐degree‐of‐freedom hybrid nanogenerator that integrates multi‐directional wave energy into a unified output, achieving 1 + 1 > 2 energy harvesting.

In this work, we propose a high‐power hybrid nanogenerator (HP‐HNG) featuring multi‐degree‐of‐freedom mechanical coupling. The HP‐HNG comprises a transmission system and power generation modules (PGMs), each containing an EMG pumping TENG, a rotational EMG (R‐EMG), and two rotational TENGs (R‐TENGs). Its motion modes are categorized as heave and pitch, with the single‐degree‐of‐freedom outputs from both modes combined through a planetary carrier in the planetary gear system. The combined energy output from heave and pitch motion is less than the sum of their individual outputs. At a generator rotational speed of 250 rpm, the peak power density of the HP‐HNG is 127.303 W m^−^^3^. After 100 h of continuous operation of the HP‐HNG, the current decay of the R‐TENG was less than 5.9%, demonstrating the HP‐HNG's excellent durability. In addition, the HP‐HNG can be used as a power source for submarine cable monitoring to prevent submarine cable failures. This work demonstrates that the HP‐HNG can simultaneously harvest wave energy from multi‐degree‐of‐freedom systems, which is conducive to promoting the practical application of blue energy and providing a sustainable energy supply for future monitoring of submarine cables and distributed sensing systems in the marine Internet of Things.

## 2 Results and Discussion

### 2.1 Structural Design and Working Principle of the HP‐HNG

We propose utilizing the HP‐HNG network, which can harvest low‐frequency continuous energy into electrical energy. Figure 1a shows the application scenario of the HP‐HNG in harvesting wave energy. Submarine cable incidents occur frequently, and fault locations are highly random, necessitating distributed condition monitoring. Multiple HP‐HNG units can be networked and deployed near islands, or oil rigs can provide power for devices such as sensors, which can be used to monitor the temperature and positioning of submarine cables.

**FIGURE 1:**
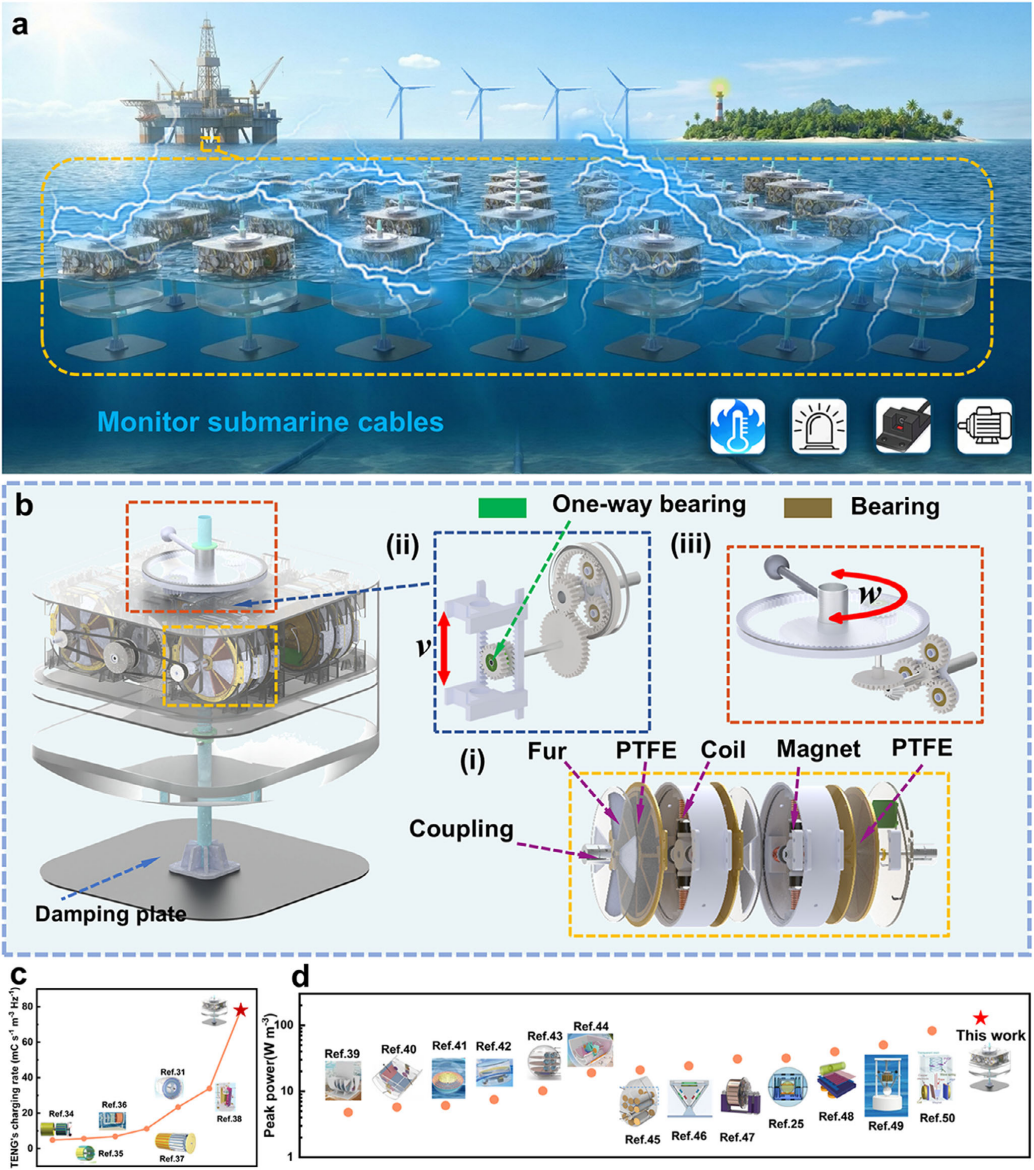
Structural design of the HP‐HNG for water waves. (a) Application scenarios of the HP‐HNG. The conceptual design of the rendered images of the drilling platform, island, and the HP‐HNG array shown in Figure 1a was developed with the assistance of Gemini 3.2 (January 2026); all final images underwent extensive manual modification and integration by the authors. The HP‐HNG structure itself is an original design by the authors. (b) Schematic illustration of the HP‐HNG, which consists of (i)R‐TENG, R‐EMG, and pump EMG and M‐TENG. The two types of motion of HP‐HNG: (ii)heave and (iii)pitch. (c) Comparison of charging rate of the R‐TENG with other TENG structures. (d) Comparison of peak power density between HP‐HNG and other hybrid nanogenerators.

Figure 1b illustrates the basic structure of the HP‐HNG, which consists of a transmission mechanism, (i) four PGMs, a float, and damping plates. Each PGM consists of one M‐TENG driven by an EMG pump, one R‐EMG, and two R‐TENGs. The motion forms of the HP‐HNG are classified as (ii) heave and (iii) pitch. The multi‐degree‐of‐freedom water wave energy is converted into unidirectional rotational mechanical energy via a transmission mechanism, which then transfers the mechanical energy to the PGMs through a synchronous belt. The positions of the transmission mechanism and PGMs of the HP‐HNG are shown in Figure S1. The HP‐HNG exhibits a fundamentally symmetric structure, with the motion directions of modules 1 and 4 being opposite to those of modules 2 and 3. The physical picture of the HP‐HNG is shown in Figure S2. As depicted in Figure S3, the transmission system consists of gears, racks, and a pendulum ball.

One‐way bearings α and β are positioned between shaft 14 and gears 12 and 13, transmitting torque clockwise in the direction parallel to the one‐way bearings. Through the HP‐HNG pitch motion, the pendulum a is driven to swing, which in turn causes gear 11 or gear 13 to drive shaft 14 to rotate unidirectionally. Similarly, the gear‐rack mechanism that harvests the heave energy uses one‐way bearings γ and δ installed between gears 8, 9, and shaft 15. With the relative motion of the gear and rack, shaft 15 rotates unidirectionally. Then, these two input motions are combined through a differential gear system, with the planetary carrier H serving as the output and connected to the synchronous wheel. The synchronous wheel transmits motion to the rotor in the PGM through belt drive. The ocean floating system has four PGMs, each of which can operate independently. If one PGM assembly fails, the others can continue to supply power. The specific motion of the HP‐HNG and its corresponding dynamic model are illustrated in Note S1.

Using Note S2 calculations, the rotational speeds of planetary carriers *H* and *H*′ can be determined. Since *n_H_* and *n_c_* are driven by a synchronous belt with a transmission ratio of 1/2, the rotational speed of *n_c_* is:

(1) $$n_{c}=4n_{a}+\frac{24v_{r}}{\pi }$$

where *n_a_*: the pendulum rotation speed, *v_r_*: the rack movement speed, *n_c_*: the rotational speed within the generator assembly.

The HP‐HNG float is the primary structure interacting with waves, and its shape significantly impacts the power generation capacity and lifespan of the entire system. The HP‐HNG float uses a spherical cylindrical structure, which provides good strength. The cylindrical shape allows the surface to absorb the distributed pressure, while the spherical bottom can effectively counteract the distributed lateral and bottom water pressure. The transmission system is installed inside the floating body, which prevents it from coming into contact with corrosive seawater. Two windows can be installed on the top or side of the HP‐HNG, facilitating the inspection and maintenance of the transmission system and PGMs.

The charging speed of the multi‐degree‐of‐freedom R‐TENG harvesting wave energy is 78 mC s^−^^1^ m^−^^3^ Hz^−^^1^, compared with previously reported TENGs [31, 34, 35, 36, 37, 38], as shown in Figure 1c. The overall charging rate of the HP‐HNG is 1.87 C s^−^^1^ m^−^^3^ Hz^−^^1^. Furthermore, power density and power, as the most important fundamental parameters, are regarded as indicators for evaluating the output performance of generators [25, 39, 40, 41, 42, 43, 44, 45, 46, 47, 48, 49, 50]. The peak power density of the HP‐HNG reaches 127.303 W m^−^^3^, as illustrated in Figure 1d and Table S1. As shown in Figure S4, compared with other hybrid nanogenerators, the peak power of the HP‐HNG is significantly increased, reaching 0.764 W, demonstrating the capability of the HP‐HNG in harvesting and converting ocean waves.

Figure 2a illustrates the detailed working principle of the M‐TENG. The Polytetrafluoroethylene (PTFE) film covering the M‐TENG serves a dual purpose of insulation and oxidation protection. The EMG pump continuously injects charge, and according to the principle of electrostatic induction, the corresponding output electrode generates opposite charge. When the rotor rotates, the electrostatic equilibrium is disrupted, generating a potential difference. The induced charge in the output electrode flows in correspondence with the bound charge in the input electrode. Therefore, there is a periodic transfer of charge between output electrodes 1 and 2, which generates an alternating current in the external load.

**FIGURE 2:**
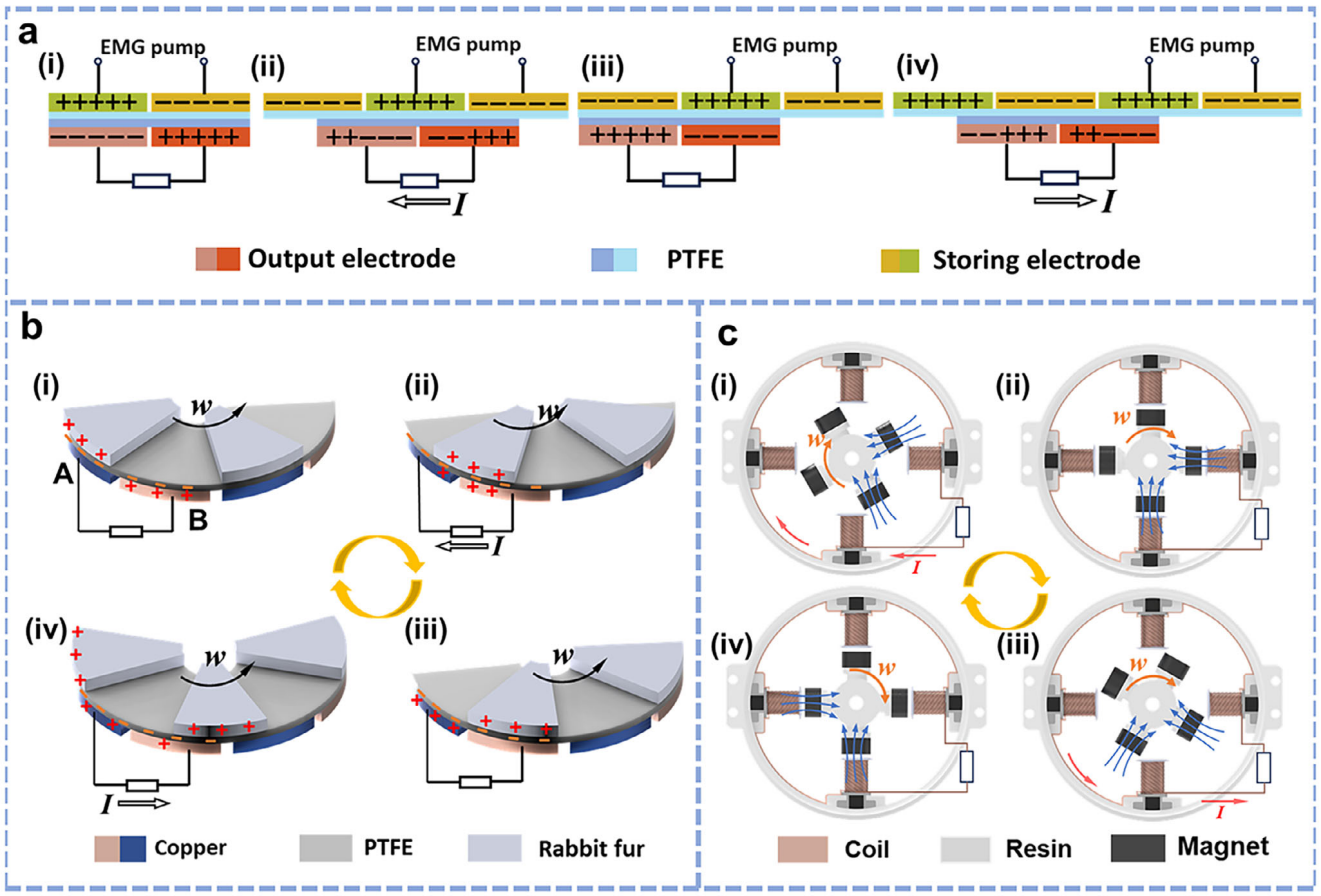
The working principles of each part in the HP‐HNG. (a) Illustration of the charge transfer process from the EMG pump to the M‐TENG. (b) The working principles of the R‐TENG. (c) The working principles of the R‐EMG.

The working principle of R‐TENG is shown in Figure 2b. PTFE film is affixed to the copper electrodes, and rabbit fur slides between two adjacent copper electrodes A and B. After several rounds of friction, the rabbit fur becomes positively charged by rubbing against the PTFE film. When the rabbit fur overlaps with electrode A (Figure 2b–i), an equal amount of negative charge is generated on the surface of the PTFE membrane. As the rabbit fur slides toward electrode B, electrons flow from electrode B to electrode A through the external circuit, balancing the change in potential difference (Figure 2b–ii). An induced current then flows from electrode A to electrode B until the rabbit fur completely overlaps electrode B (Figure 2b–iii). As the rabbit fur slides further, electrons on electrode A flow back to electrode B (Figure 2b–iv), inducing a reverse current. The periodic structure of the TENG means that continuous movement of rabbit fur generates an alternating output. The working principle of EMG is shown in Figure 2c. In the initial state, the magnetic flux within the coil remains unchanged, so no current flows through the coil, as shown in Figure 2c(i). When the magnet approaches the coil, the increase in magnetic flux induces a current, as depicted in Figure 2c(ii). When the magnet aligns with the coil, as shown in Figure 2c(iii), the magnetic flux within the coil remains constant, and no current is generated. Finally, when the magnet moves away from the coil, the direction of current flow reverses compared to Figure 2c(ii), as depicted in Figure 2c(iv). Four coils are connected in series with identical winding directions to achieve voltage superposition in the EMG.

### 2.2 Output Performance of the M‐TENG Module

Figure 3 demonstrates the output performance of the EMG pump and the M‐TENG. To investigate their output characteristics, a rotating motor was employed as the external excitation source in the experiment. Figure 3a and Figure S5 present the schematic diagram and physical image of the EMG pump‐M‐TENG structure. The EMG supplies charge to the M‐TENG via an electrical circuit. The circuit diagram connecting the EMG and the M‐TENG is shown in Figure 3b. The circuit primarily consists of four modules: rectification, oscillation, feedback transformer, and boost circuits. At a rotational speed of 250 rpm and an 8x voltage multiplication ratio, the optimal values for resistances *R*_1_ and *R*_2_ were determined to be 27 KΩ and 0 Ω, respectively (Figure S6). Among the 1–4 transformers, the turns ratios of the primary, secondary, and feedback coils vary for each transformer. As shown in Figure 3c, under 12 fold voltage multiplication, the circuit incorporating Transformer 3 in the 1–4 transformer configuration produced the highest output current of 127.21 µA. The voltage and current generated by the EMG pump at different rotational speeds are illustrated in Figure 3d and Figure S7. At a rotational speed of 250 rpm, the EMG pump's output voltage reached 1584 V within 5 s. As shown in Figure 3e, compared to a 2x voltage multiplier circuit, the EMG pump's output voltage is essentially 2n times that of the 2x circuit (where n ≥ 2 and n represents the capacitor count). Since the HP‐HNG contains four PGMs, each equipped with an EMG pumping M‐TENG, it incorporates four voltage‐doubling circuit boards. The voltage output from each board remains essentially constant (Figure S8).

**FIGURE 3:**
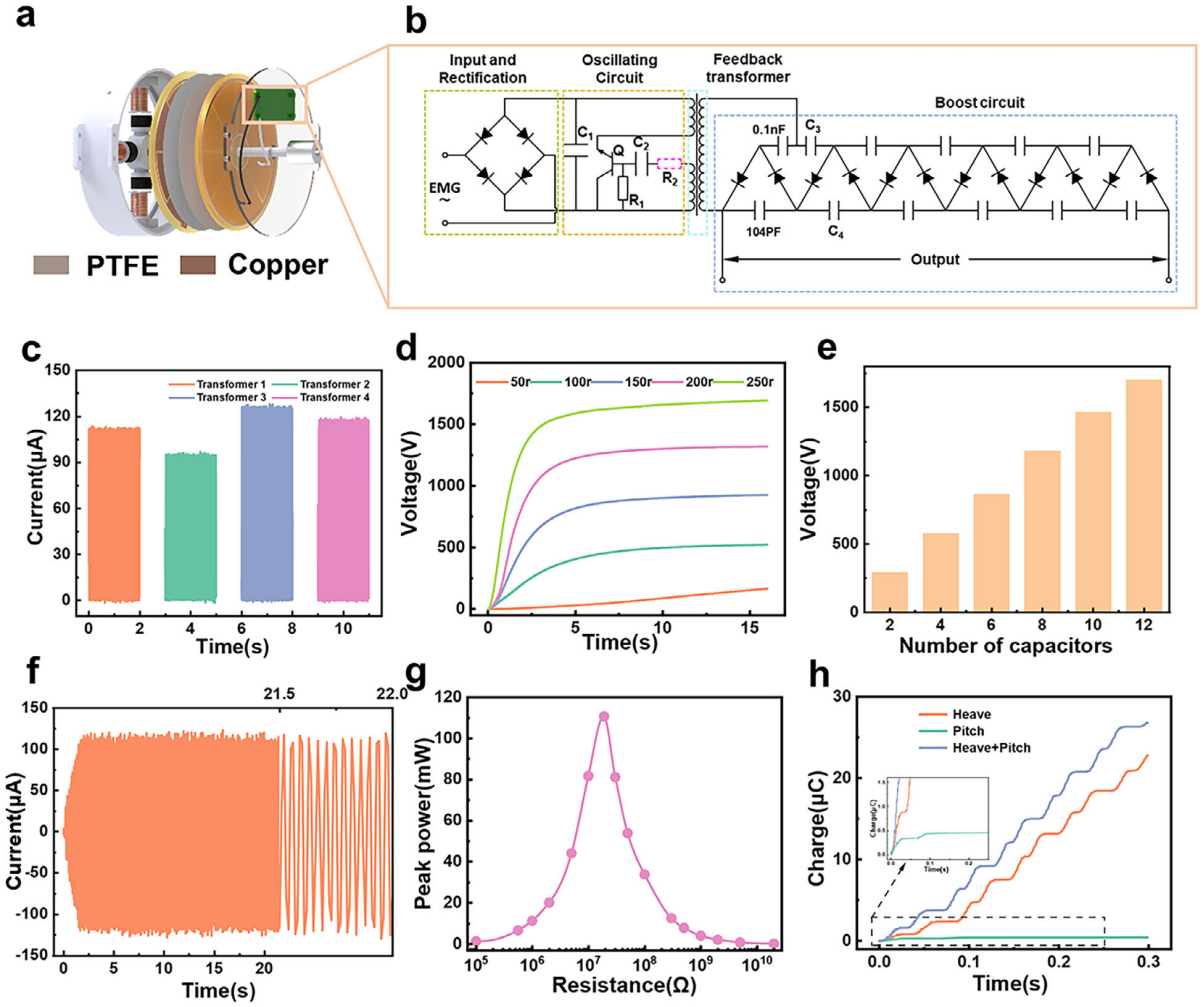
Output Performance of the M‐TENG. (a) The positional relationship between the EMG pump and the M‐TENG. (b) Circuit diagram of the EMG pump. (c) Transformer selection in the circuit. (d) Output voltage of the EMG pump at different rotational speeds. (e) The effect of capacitor quantity on pump output voltage. (f) *I_sc_* of the M‐TENG. (g) The peak power of the M‐TENG at 250 rpm. (h) Charge transfer rate at 1 Hz frequency during heave, pitch, and combined heave+ pitch.

Figure 3f and Figure S9 illustrate the short‐circuit current (*I_sc_*), transferred charge (*Q_sc_*), and open‐circuit voltage (*V_oc_*) of the M‐TENG at a rotational speed of 250 rpm. As the charge generated by the EMG pump is rapidly injected into the storage electrode of the M‐TENG via the voltage‐doubling circuit board, the charge density within the storage electrode approaches saturation. The M‐TENG output first rises rapidly, then stabilizes at values of 121.13 µA, 0.39 µC, and 1.52 kV, exhibiting a trend consistent with the charge pumping mechanism. Subsequently, at a rotational speed of 250 rpm, the peak power curve of the M‐TENG under different resistances is measured (Figure 3g). At the same time, the curves of *I_sc_* and *V_oc_* with resistance are obtained (Figure S10). With a matching resistance of 20 MΩ, the maximum output power can reach 110.65 mW, demonstrating excellent energy harvesting capability. As shown in Figure 3h, when the water wave frequency is 1 Hz and the amplitude is 20 cm, the HP‐HNG undergoes heave, and the EMG pump outputs a charge of 22.778 µC within 0.3 s. When the HP‐HNG undergoes pitch, the EMG pump outputs a charge of 0.455 µC within 0.3 s. When the HP‐HNG undergoes heave + pitch, the EMG pump outputs 26.773 µC within 0.3 s. Comparing these charge outputs reveals that during the HP‐HNG's multi‐degree‐of‐freedom motion, the EMG pump's output charge exceeds the sum of outputs during heave or pitch, achieving a synergistic effect where the EMG pump's output charge demonstrates a 1 + 1 > 2 advantage.

### 2.3 Output Performance of the R‐TENG and the R‐EMG Module

Figure 4 illustrates the output performance of R‐EMG and R‐TENG. The HP‐HNG comprises four PGMs, each containing two R‐TENG units and one R‐EMG unit, numbered 1–8 and 1–4, respectively. Figure 4a and Figure S11 present schematic diagrams and physical images of the R‐TENG‐R‐EMG‐R‐TENG structure. The distance between the magnet rotor and coil in the R‐EMG is 2 mm. The coil arrangement of the R‐EMG is shown in Figure S12. The *V_oc_* and *I_sc_* of R‐EMG are proportional to rotational speed (Figure S13). At different rotational speeds (r = 50, 100, 150, 200, and 250 rpm), *V_oc_* increases from 2.4 to 10.4 V, while *I_sc_* rises from 2.05 to 9.07 mA. As shown in Figure 4b, the *V_oc_* of each R‐EMG remains essentially consistent at a rotational speed of 250 rpm. As shown in Figure S14, the peak power of the R‐EMG increases with rotational speed, while the optimal load resistance remains constant at 1200 Ω. As depicted in Figure 4c and Figure S15, R‐EMG1, 2, 3, and 4 are connected in series. The output currents of R‐EMG1‐2, R‐EMG1‐3, and R‐EMG1‐4 remain essentially unchanged, while the voltage of R‐EMG1 + 2 + 3 + 4 is 3.82 times that of R‐EMG1. As shown in Figure 4d, the peak powers of R‐EMG1, R‐EMG1‐2, R‐EMG1‐3, and R‐EMG1‐4 matched resistors exhibit peak powers of 22.85 mW (1.2 KΩ), 45.61 mW (2.2 KΩ), 65.05 mW (3.3 KΩ), and 93.22 mW (4.5 KΩ), respectively.

**FIGURE 4:**
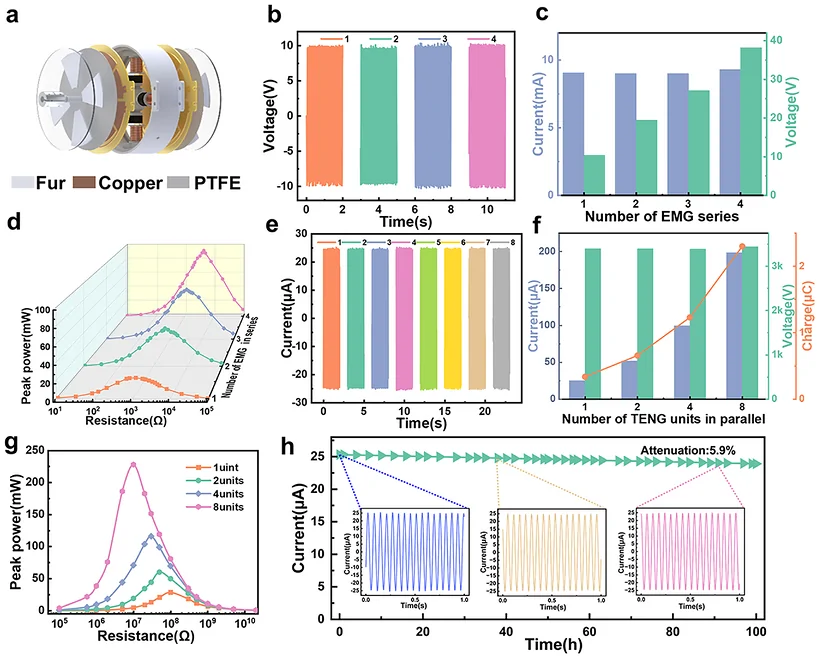
Output Performance of the R‐TENG and the R‐EMG in the HP‐HNG. (a) The positional relationship between the R‐TENG and the R‐EMG. (b) *V_oc_* of each R‐EMG at 250 rpm. (c) Relationship between the number of R‐EMGs in series and *V_oc_* / *I_sc_*. (d) The relationship between the peak power curve and the number of R‐EMGs. (e) *I_sc_* of each R‐TENG at 250 rpm. (f) Relationship between the number of R‐TENGs in parallel and *I_sc_*, *V_oc_*, and *Q_sc_*. (g) The peak power‐load curve for paralleled R‐TENG units. (h) Current stability test of R‐TENG running for 100 h.

Figure S16 shows the output comparison using PTFE as the positive charge material and four different materials as the negative charge material, with the speed controlled at a rotational speed 250 rpm. Testing revealed that 3 mm thick rabbit fur is the optimal negative charge material, enabling the R‐TENG to achieve an output voltage of approximately 3.34 kV. Figure S17 shows the output performance of the R‐TENG at different rotational speeds. Theoretical analysis indicates that the I_SC_ increases with speed, as depicted in Figure S17a. As shown in Figure S17b, the *V_oc_* remains unaffected by speed as long as the device structure and initial surface charge density remain constant. As shown in Figure S17c, the *Q_sc_* reaches a balanced state at the saturation point of the PTFE film. As shown in Figure 4e, when the R‐TENG rotates at 250 rpm, the *I_sc_* of each R‐TENG is basically the same. As shown in Figure 4f and Figure S18, at a rotational speed of 250 rpm, the *I_sc_* increases from 25.3 to 198.5 µA with the number of parallel R‐TENG units, while the *V_oc_* remains essentially unchanged. The *Q_sc_* increases from 0.339 to 2.301 µC. As shown in Figure 4g, at a rotational speed of 250 rpm, the peak power increased from 28.39 to 228.01 mW with the number of parallel R‐TENG units, while the optimal load resistance decreased from 100 to 10 MΩ. To verify the durability of the R‐TENG, a long‐term endurance test was conducted on the R‐TENG basic unit at a rotational speed of 250 rpm, as shown in Figure 4h. After 100 h of continuous operation, the output current of the R‐TENG decreased from 25.3 to 23.8 µA, representing an attenuation of <5.9%, demonstrating its significant advantage in durability. The high durability of the R‐TENG basic unit can also be verified by scanning electron microscopy (SEM) observation of the PTFE surface (Figure S19). Due to the low sliding friction resistance of rabbit hair on PTFE, wear on the PTFE surface is extremely minimal. As shown in Figure S20, the maximum total average power for M‐TENG, R‐TENG, and R‐EMG are 222.8, 111, and 45.9 mW, respectively. The HP‐HNG can generate a peak power and average power of 0.764 and 0.379 W, corresponding to a peak power density and average power density of 127.303 and 63.28 W m^−^^3^, respectively.

### 2.4 Output Performance of Each Module of the HP‐HNG in Water Waves

The HP‐HNG exhibits multidirectional motion within water waves. Beyond kinetic energy propagating along the wave direction, the waves also harbor substantial gravitational potential energy in the vertical direction, as illustrated in Figure 5a and Figure S21. The equations of motion are shown in Note S3. The energy‐harvesting module of the HP‐HNG, which harvests energy through pitch motion, is isolated from the system and simplified as shown in Figure S21a.

(2) $$\ddot{\theta }+\frac{2b}{m_{1}l^{2}+2a}\theta +\frac{m_{1}l}{m_{1}l^{2}+2a}\left(h\ddot{\varphi }+{\dot{\varphi }}^{2}\cos\theta +g\sin\varphi \right)\;\sin\theta =0$$

where φ is the tilt angle of the entire device; θ is the angle of oscillation of the pendulum ball around its central axis; *l* is the radius of rotation; *m*_1_ is the mass of the pendulum ball; *a* is the viscous mass coefficient; *b* is the viscous damping coefficient; *h* is the distance from the pendulum ball to the plate.

**FIGURE 5:**
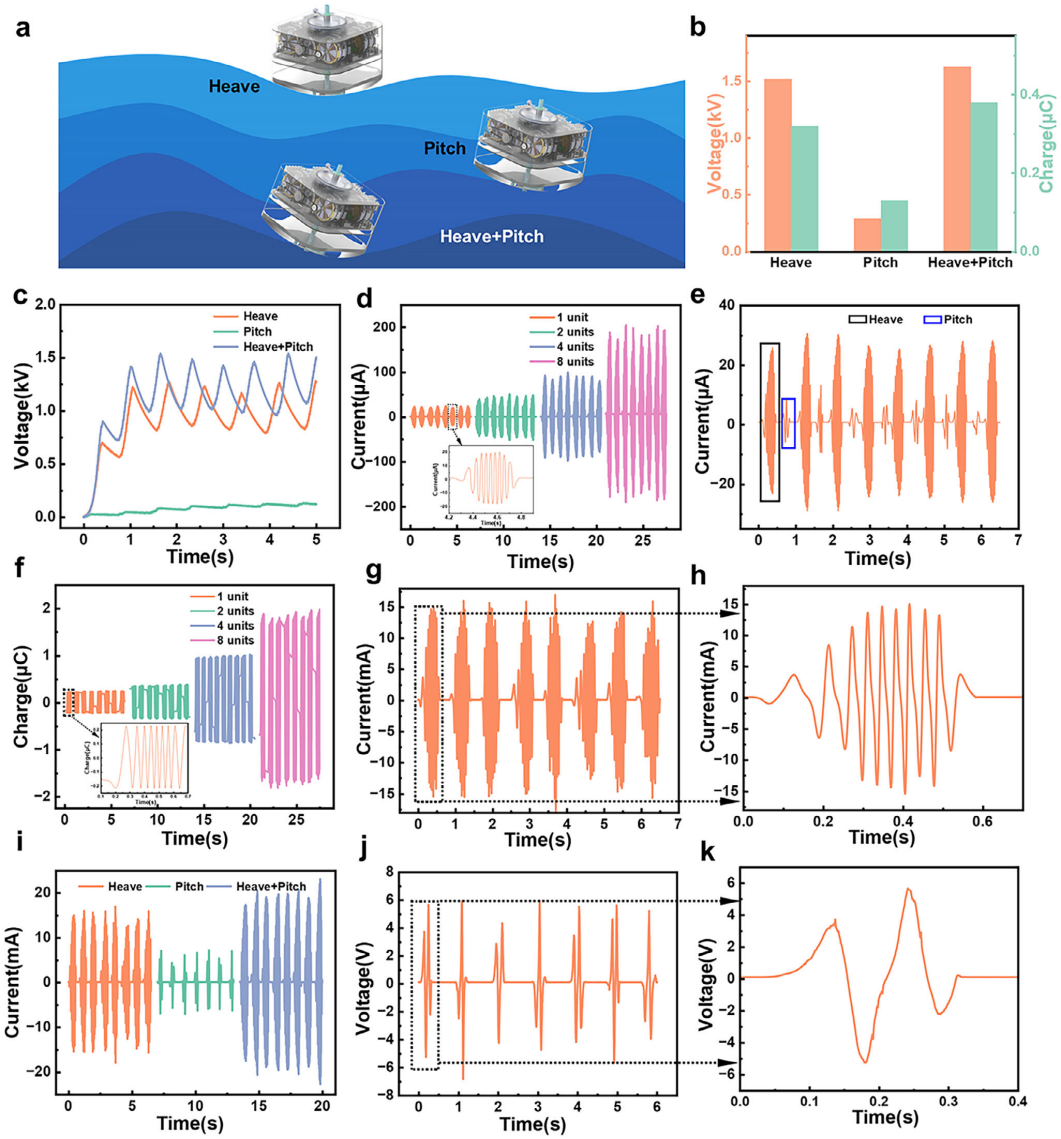
Output performance of each module in the HP‐HNG under water waves. (a) Three different motion states of the HP‐HNG in water waves. (b) The relationship of *V* _*OC*_and *Q_SC_* of the M‐TENG in heave, pitch, and heave + pitch at a water wave frequency of 1 Hz and an amplitude of 20 cm. (c) Voltage variation curves of the EMG during heave, pitch, and combined heave + pitch motions of the HP‐HNG. (d) Relationship between the number of R‐TENGs and their current output when the HP‐HNG undergoes heave motion under a water wave frequency of 1 Hz and an amplitude of 20 cm. (e) Current variation curves of the R‐TENG during heave and pitch motions when they occur separately. (f) Relationship between the number of R‐TENGs and their charge output when the HP‐HNG undergoes heave motion under a water wave frequency of 1 Hz and an amplitude of 20 cm. (g) Current variation curve of the R‐EMG when the HP‐HNG undergoes heave motion under a water wave frequency of 1 Hz and an amplitude of 20 cm. (h) Enlarged view of the R‐EMG current.(i) Current variation curves of the R‐EMG during heave, pitch, and combined heave + pitch motions of the HP‐HNG.(j) Current variation curve of the R‐EMG when the HP‐HNG undergoes pitch motion.(k) Enlarged view of the R‐EMG voltage.

Coupled equation for float heave + pitch:

(3) $$\begin{array}{ccc} & & \left(m+m_{a}+m_{2}\right)\ddot{x}+\left(c_{1}+c_{a}\right)\dot{x}\\ & & \;+\;\left(k_{a}+k_{1}+k_{2}\right)x=F_{1}\;\cos\left(\omega t\right) \end{array}$$

(4) $$\begin{array}{ccc} & & \left(I_{2}+A_{2}\right)\ddot{\varphi }\left(t\right)+c_{2}\dot{\varphi }\left(t\right)+\rho gV_{0}\;\varphi \left(t\right)\\ & & \;-\;k_{3}\varphi \left(t\right)x\;\left(t\right)=M_{1}\;\cos\left(\omega t\right) \end{array}$$

where *m* of the HP‐HNG mass; *m*_2_ of the additional mass; *m*_a_ of the equivalent mass; *c*_1_ of the additional damping; *c*_a_ of the equivalent damping; *k*_a_ of the pitching restoring stiffness; *k*_1_ of the upper spring stiffness; *k*_2_ of the lower spring stiffness; *F*_1_ of the force driving module transmission in the float; *I*_2_ of the pitching moment of inertia of the float; *A*_2_ is the additional moment of inertia during heave; *c*_2_ is the heave damping coefficient; *M*_1_ is the amplitude of the heave wave moment.

When the wave frequency is 1 Hz, and the amplitude is 20 cm, the rotor in the HP‐HNG completes approximately 4 revolutions in 1 s. The rotor speed and frequency in the laboratory are essentially consistent, with an average value of about 250 rpm. As shown in Figure 5b and Figure S22, during heave of the HP‐HNG, the M‐TENG recorded voltages, charges, and currents of 1.52 kV, 0.32 µC, and 101.69 µA, respectively. During heave motion, the M‐TENG recorded voltages and charges of 0.29 kV and 0.13 µC, with a corresponding current of 22.12 µA. During the HP‐HNG heave + pitch motion, the M‐TENG voltage and charge were 1.63 kV, 0.38 µC, and 121.32 µA, respectively. The HP‐HNG heave exhibits second‐order forced vibration, where velocity peaks when acceleration is zero and approaches zero when acceleration is maximum. The instantaneous maximum speed of the generator rotor reached 500 rpm. As shown in Figure 5c and Figure S23, during the HP‐HNG's heave motion, pitch motion, and combined heave + pitch motion, the EMG pump voltage after 5 s of motion was 1.28, 0.13, and 1.51 kV, respectively; the corresponding currents were 0.62, 0.18, and 0.76 mA. When the wave frequency remains constant, the R‐TENG can stably harvest wave energy across an angle range from 0° to 330°, with its output performance nearly unaffected by the change in wave direction (Figure S24). When the rotor speed of the R‐TENG reaches its maximum, the current of the R‐TENG also reaches its maximum. As shown in Figure 5d and Figure S25a, when the HP‐HNG undergoes heave and pitch motions, the current through the R‐TENG increases as the number of parallel R‐TENG units increases. As depicted in Figure 5e, heave and pitch motions occur sequentially, with the HP‐HNG first experiencing heave followed by pitch. The velocity generated by the HP‐HNG's heave motion exceeds that produced by its pitch motion. The charge quantity in the R‐TENG does not vary with velocity changes. As shown in Figure 5f and Figure S25b, during the heave and pitch motions of the HP‐HNG, the *Q_sc_* quantities increase from 0.268 µC and 0.212 µC to 1.954 µC and 1.554 µC, respectively, as the number of parallel R‐TENG units increases. As shown in Figure S25c, when heave and pitch occur simultaneously, taking the charge transfer of one R‐TENG as an example, the following equation holds:

(5) $$Q_{H+P}>max\left(Q_{H},Q_{p}\right)$$

where *Q*_*H* + *P*_: Charge transferred by heave + pitch motion of the R‐TENG; *Q_H_*: Charge transferred by heave of the R‐TENG; *Q_p_*: Charge transferred by pitch motion of the R‐TENG.

Furthermore, as shown in Figure S26, the *V_oc_* remains essentially unchanged with an increase in the number of R‐TENG units connected in parallel. The *V_oc_* of the R‐TENG is largely independent of the operational state of the HP‐HNG.

At a water wave frequency of 1 Hz and an amplitude of 20 cm, since the heave motion of the HP‐HNG is a variable‐speed motion, the peak values of R‐EMG current and voltage continuously fluctuate. As shown in Figure 5g,h, during the HP‐HNG heave motion, the current peak is approximately 15 mA. The voltage and current of R‐EMG are related to rotor speed. When the instantaneous rotor speed reaches its maximum, the R‐EMG current and voltage attain their peak values. The maximum instantaneous velocity of the HP‐HNG heave occurs at half of the HP‐HNG heave motion cycle. As shown in Figure S27a, during the HP‐HNG's pitch motion, the R‐EMG current is approximately 6 mA. During the HP‐HNG heave + pitch motion, the R‐EMG current can reach 20 mA (Figure S27b). Figure 5i displays the current variation curve of an R‐EMG unit during the HP‐HNG heave, pitch, and heave + pitch motions. Concurrently, as shown in Figure S28a, the R‐EMG voltage peaked at approximately 15 V during the HP‐HNG heave. During the HP‐HNG pitch, the R‐EMG voltage was approximately 6 V (Figure 5j,k). When the HP‐HNG underwent heave + pitch, the R‐EMG voltage reached around 20 V (Figure S28b). Figure S28c displays the voltage variation curve of an R‐EMG unit during the HP‐HNG's heave motion, pitch motion, and combined heave + pitch motion. The HP‐HNG's movement through water waves is non‐uniform, unlike laboratory motor‐driven motion. Consequently, the peak values of certain velocity‐related variables exceed those observed during uniform laboratory motion.

### 2.5 Output Performance of the HP‐HNG in Water Waves

We have developed an efficient integrated self‐powered energy harvesting system to effectively harvest wave energy. The connection methods of M‐TENG, R‐EMG, and R‐TENG in the HP‐HNG are displayed in Figure S29. To achieve the broad application of the HP‐HNG in monitoring islands, offshore drilling platforms, and submarine cables, we adopted an efficient self‐powered energy management circuit to manage the harvested energy. As shown in Figure S30, the circuit includes R‐TENG1‐8, M‐TENG1‐4, R‐EMG, a rectifier bridge, LTC‐3588, and a classic buck converter circuit. When the voltage across *C_0_* exceeds the threshold voltage of the GDT switch during operation, the GDT switch turns on, instantly releasing the stored energy from capacitor *C_0_* into the inductor (*L*). Subsequently, the GDT turns off, and the energy in the inductor continues to charge the capacitor through the closed‐loop circuit at the rear end, forming a complete energy cycle. Closing switch *Q* stores electrical energy in the rear capacitor *C*_1_through the preceding circuit and powers the 3588 chips via *C*_1_, enabling the chip to operate. The 3588 chips can continuously and stably output a voltage of 2.5 V at the output end, meeting the working conditions of the sensor and allowing the sensor to operate. As shown in Figure 6a and Figure S31, when the rotor speed is 250 rpm, the optimal *L*, gas discharge tube (GDT) breakdown voltage, and capacitance *C_0_* value can be determined using the control variable method. These values are 10 mH, 2 kV, and 0.1 nF, respectively. When using a power management circuit (PMC), R‐TENG charges a 1 mF commercial capacitor from 0 µC to 786.5 µC within 5 s. In contrast, without PMC, the charge reaches only 30.6 µC in the same timeframe, representing a charging efficiency improvement of approximately 28.3 times (Figure S32). Figure S33a shows the charging curve for one module charging different capacitors at a rotor speed of 250 rpm. Within 5 s, one module can charge a 10 mF capacitor to 1.144 V. The voltage curve for charging a 2.2 mF capacitor through the PMC at different rotor speeds was also measured (Figure S33b). At 250 rpm, a single module with the PMC could charge the 2.2 mF capacitor to 3.2 V within 5 s.

**FIGURE 6:**
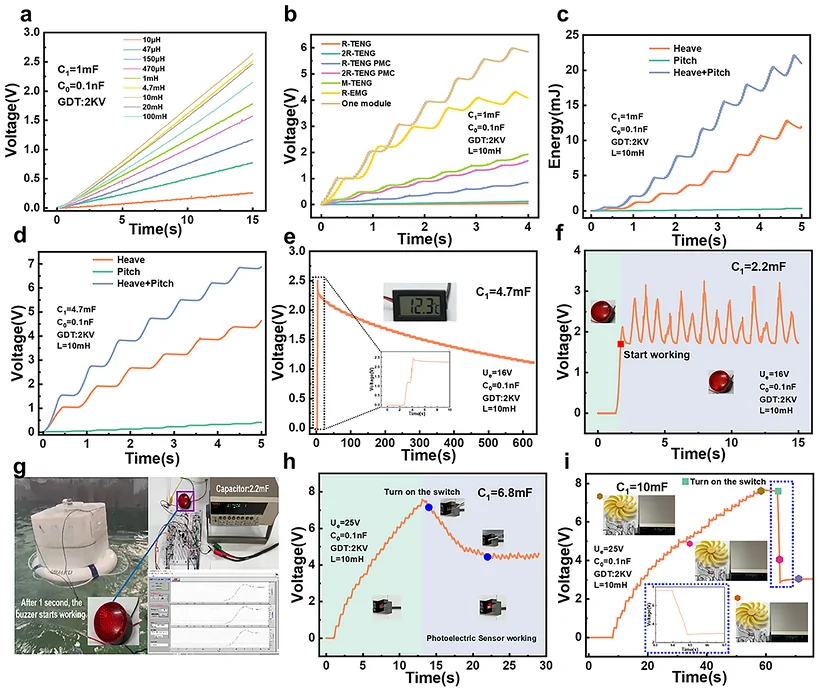
Performance demonstration of the HP‐HNG as a power source. (a) Selection of the inductance L for the management circuit. (b) Charging curves for different sections within a single module when the device undergoes heave + pitch in a water wave environment. (c) Energy charging curves of a single module powering a capacitor under different motion modes in a water wave environment at 1 Hz and an amplitude of 20 cm. (d) Charging curves of the HP‐HNG under different motion modes in a water wave environment. (e) Self‐powering of a thermometer by the HP‐HNG in a water wave environment. (f) Self‐powering of a buzzer by the HP‐HNG in a water wave environment. (g) Physical image of the HP‐HNG self‐powered buzzer at a water wave frequency of 1 Hz and the amplitude of 20 cm. (h) Powering a photoelectric sensor by the HP‐HNG in a water wave environment. (i) Driving a small motor by the HP‐HNG in a water wave environment.

Figure 6b shows the charging voltage curves for R‐TENG, 2R‐TENG, R‐TENG PMC, 2R‐TENG PMC, M‐TENG, R‐EMG, and a single module charging a 1mF capacitor when the wave frequency is 1 Hz, the amplitude is 20 cm, and the HP‐HNG undergoes heave + pitch motion. During the early charging phase, EMG charges faster than TENG but quickly saturates due to its lower *V_oc_*. In contrast, although TENG charges more slowly, the capacitor can sustain charging to higher voltages. After 4 s of charging, the voltages reached 0.043, 0.123, 0.842, 1.679, 1.927, 4.089, and 5.856 V for R‐TENG, 2R‐TENG, R‐TENG PMC, 2R‐TENG PMC, M‐TENG, R‐EMG, and a single module, respectively. Through the power management circuit, the charging rate of the R‐TENG increased from 0.011 to 0.211 V/s, representing a 19.14 fold improvement in charging speed. As shown in Figure S33, the charging curves for the 1 mF capacitor by different sections and the entire assembly of a single module during the HP‐HNG's heave and pitch motions, respectively. Therefore, the capacitor charging formula is derived as:

(6) $$U_{H+P}>U_{H}+U_{P}$$

where *U*_*H* + *P*_ is the charging voltage of a single module during heave + pitch motion;

*U_H_* is the charging voltage of a single module during heave motion;

*U_P_* is the charging voltage of a single module during pitch motion.

As shown in Figure 6c, during 5 s of the HP‐HNG heave + pitch motion, a single module generates approximately 21.5 mJ of energy. This exceeds the combined energy produced by the HP‐HNG during separate heave and pitch motions.

According to the formula:

(7) $$E_{C_{1}}=\frac{1}{2}\;C_{1}U^{2}$$

where $E_{C_{1}}$: the stored energy in the capacitor; *C*_1_: the capacitance of the capacitor; *U*: the voltage across the capacitor.

At a water wave frequency of 1 Hz and an amplitude of 20 cm, the HP‐HNG's heave, pitch, and combined heave + pitch motions over 5 s can charge a 4.7 mF capacitor to 4.644, 0.411, and 6.875 V, respectively (Figure 6d). Figure S35 demonstrates that during the combined heave + pitch motion, the HP‐HNG generates 111.06 mJ of energy in 5 s. After 100 h of continuous operation, the output current of the HP‐HNG decreased from 111.06 to 102.6 mJ, representing an attenuation of <7.7%, demonstrating its significant advantage in durability. In addition, the charging energy of HP‐HNG under different water wave frequencies within 5 s is measured. When the water wave increased from 0.2 to 1 Hz, the energy generated by HP‐HNG increased from 7.54mJ to 111.06 mJ (Figure S36).

As shown in Figure 6e and Movie S1 using the HP‐HNG to charge a 4.7mF capacitor for approximately 2 s, the capacitor voltage can reach 2.4 V, and enabling the thermometer to operate for 630 s, which is sufficient to continuously monitor the temperature of submarine cables (Tested in the wave pool at the Beijing Institute of Nanoenergy and Nanosystems, Chinese Academy of Sciences. The size of the wave pool is 45 × 4 × 3m^3^.). Furthermore, in emergency situations, the HP‐HNG can autonomously activate a buzzer to alert workers. When the water wave frequency is 1 Hz and the amplitude is 20 cm, the buzzer begins to operate after the HP‐HNG moves for 1.7 s (Figure 6f and Movie S2). When the water wave frequency is 1 Hz and the amplitude is 20 cm, the physical image of the HP‐HNG self‐powered buzzer is shown in Figure 6g. As the HP‐HNG continues to move, the buzzer can keep working, demonstrating the application of the HP‐HNG in self‐powered sensing. The submarine environment is complex, and submarine cables may suffer unexpected damage; therefore, monitoring these cables is essential. The HP‐HNG can also drive an optoelectronic sensor to monitor any displacement of the submarine cables. As shown in Figure 6h, the HP‐HNG moves for 13.2 s, charging a 6.8 mF capacitor to 7.6 V, then after turning on switch *Q*, the optoelectronic sensor can operate continuously, and the HP‐HNG continues to move, demonstrating that after providing an initial voltage to the capacitor, the HP‐HNG can achieve self‐driven sensing (Movie S3). In addition, the HP‐HNG can also use large capacitors to store energy to drive micro motors. The rated current of micro motors is 20 mA, and it is difficult to drive micro motors using a traditional TENG to charge capacitors, which demonstrates the advantage of using multiple energy harvesting methods to harvest wave energy. In the circuit driving the micro motor, the 3588 chip is required to maintain the output voltage at a stable value. As shown in Figure 6i, the HP‐HNG can charge a 10mF capacitor to 7.71 V within 50 s. Subsequently, switching on switch *Q* causes the small motor to rotate for 3 s before stopping, at which point the voltage across the capacitor reaches 3 V (Movie S4). A multifunctional sensor combining thermometers, a buzzer, and optoelectronic sensors can monitor submarine cable status, preventing issues that may compromise cable operation. The HP‐HNG effectively harvests low‐frequency wave energy to meet diverse power demands. Furthermore, its ability to drive micro‐motors demonstrates the potential for future hybrid nanogenerators in large‐scale wave energy harvesting applications.

## 3 Conclusion

In this work, we propose an HP‐HNG with multi‐degree‐of‐freedom mechanical coupling for harvesting vertical and longitudinal wave energy. The HP‐HNG converts multi‐directional energy into a single energy output via mechanical transmission at the rotor containing the PGMs. The sum of energy outputs from heave and pitch motions is less than the combined output of heave and pitch motions in the HP‐HNG, achieving an energy harvesting effect where 1 + 1 > 2. The EMG pump injects positive and negative charges into the M‐TENG, achieving high charge density on the M‐TENG surface and enhancing its output performance. Under matched resistances of 20 MΩ, 10 MΩ, and 4.5 KΩ, the peak powers of the M‐TENG, R‐TENG, and R‐EMG are 110.65, 228.01, and 93.22 mW, respectively. The peak power density of the HP‐HNG reached 127.303 W m^−^^3^. Under a water wave frequency of 1 Hz and an amplitude of 20 cm, the HP‐HNG delivered 111.06 mJ of energy within 5 s during heave + pitch motion. When the HP‐HNG is operating, it can supply power to sensors such as temperature sensors and buzzers to monitor submarine cables, providing guidance for ensuring power grid safety. This work provides a promising strategy for efficiently harvesting wave energy based on mechanical structures, which will play an important role in subsea monitoring and environmental sensing in the future.

## 4 Experimental Section

### 4.1 Fabrication of the HP‐HNG

The HP‐HNG module consists of a spherical float, a mechanical transmission system, and four PGMs. The effective volume of the HP‐HNG is 0.006 m^3^. All gears (photosensitive resin) in the mechanical transmission system are fabricated via 3D printing with a module of 2; specific parameters are detailed in Note S2. All transmission shafts are constructed from aluminum. The pitch transmission drives the large planetary gear. The heave transmission drives the small planetary gear. Power is ultimately output via the planetary carrier. The planetary carrier shaft and the rotor of the power generation assembly are connected via a synchronous belt drive.

### 4.2 Fabrication of the M‐TENG Module

The M‐TENG consists of five modules: EMG, circuit board, inductive electrode, PTFE, and power generation electrode. The neodymium iron boron magnets in the EMG and the induction electrode in the M‐TENG function as the rotor. The four coils in the EMG have the following specifications: (inner diameter 14 mm, outer diameter 18 mm, height 19 mm, wire diameter 0.1 mm, number of turns 2550). The angle between adjacent coils is 90°. The circuit board dimensions are: length 55 mm, width 28 mm. Both the induction electrode and the power generation electrode are fabricated using PCB technology. The induction electrode has an inner diameter of 8 mm and an outer diameter of 134 mm. The power generation electrode has an inner diameter of 12 mm and an outer diameter of 138 mm. A layer of PTFE film with a thickness of 0.08 mm is attached to each of the two electrodes.

### 4.3 Fabrication of the R‐TENG and the R‐EMG Module

The R‐TENG and R‐EMG are divided into rotor and stator modules. Rotor modules: The neodymium iron boron magnet (diameter 18 mm, thickness 10 mm) in the R‐EMG and the friction layer (rabbit hair) in the R‐TENG. Stator modules: The cylindrical coil holder has a diameter of 143.5 mm and a thickness of 38 mm. The generator electrode has an inner diameter of 12 mm and an outer diameter of 143.5 mm. Besides, a PTFE film is applied to the generator electrode.

### 4.4 Electrical Measurement and Characterization

PTFE surface morphology was measured using a scanning electron microscope (Nova Nano SEM 450, FEI). *I_sc_* and *Q_sc_* for the TENG, along with *I_sc_* and *V_oc_* for the EMG, were acquired using a programmable multimeter (Model 6514, Keithley). Acquired voltages and currents were transmitted to a computer via a signal acquisition device (USB‐6356 NI) for recording. It is important to note that the negative sign of the current is due to an inverter in the instrument's signal output port during current and capacitance testing modes. The *V_oc_* of the TENG was measured using a high‐speed electrostatic voltmeter (Trek 341B, Advanced Energy).

## Author Contributions

**Shuai Ding**: Writing – original draft, data curation, validation, software, formal analysis, visualization. **Yuanzhuo Tian**: software. **Hua Zhai**: resources, conceptualization, methodology, writing – review and editing, funding acquisition, supervision. **Xiao Long**: software, investigation. **Hao Gong**: validation, investigation. **Zhong Lin Wang**: writing – review and editing, conceptualization, methodology, supervision. **Long Zheng**: visualization. **Xucong Wang**: visualization. **Zhanyong Hong**: conceptualization. **Xiangyu Chen**: writing – review and editing, resources, funding acquisition, conceptualization, methodology, supervision.

## Conflicts of Interest

The authors declare no conflict of interest.

## Supporting information




**Supporting File 1**: advs77154‐sup‐0001‐SuppMat.docx.

**Supporting File 2**: advs77154‐sup‐0002‐MovieS1.mp4.

**Supporting File 3**: advs77154‐sup‐0003‐MovieS2.mp4.

**Supporting File 4**: advs77154‐sup‐0004‐MovieS3.mp4.

**Supporting File 5**: advs77154‐sup‐0005‐MovieS4.mp4.

**Supporting File 6**: advs77154‐sup‐0006‐SuppMat.docx.

## Data Availability

The data that support the findings of this study are available from the corresponding author upon reasonable request.
