# Supplementary material for: High‐Power Hybrid Nanogenerator With Multi‐Degree‐of‐Freedom Mechanical Coupling for Harvesting Water Wave Energy
Source: Adv Sci (Weinh). 2026 Aug 14;13(61):e77154. doi: 10.1002/advs.77154 (PMC13473930; doi:10.1002/advs.77154)
Supplement: Supplementary file 2 [file ADVS-13-e77154-s006.docx]

**Graphical Abstract**

A high-power hybrid nanogenerator (HP-HNG) with multi-degree-of-freedom mechanical coupling is reported, integrating multi-directional wave energy for output to achieve 1+1>2 energy harvesting. The HP-HNG achieves a peak output power of 0.764 W, corresponding to a peak power density of 127.303 W m⁻³. At a wave frequency of 1 Hz, the HP-HNG delivers 111.06 mJ of energy within 5 seconds under pendulum + heave motion. This work demonstrates the HP-HNG's capability to simultaneously harvest multi-degree-of-freedom wave energy, providing sustainable power for future submarine cable monitoring and distributed sensing systems in the marine Internet of Things.
