# Supplementary material for: High‐Power Hybrid Nanogenerator With Multi‐Degree‐of‐Freedom Mechanical Coupling for Harvesting Water Wave Energy
Source: Adv Sci (Weinh). 2026 Aug 14:e77154. Online ahead of print. doi: 10.1002/advs.77154 (PMC13473930; doi:10.1002/advs.77154)
Supplement: Supplementary file 1 [file ADVS-9999-e77154-s004.docx]

**Supporting Information**

**High-Power Hybrid Nanogenerator with Multi-Degree-of-Freedom Mechanical Coupling for Harvesting Water Wave Energy**

Shuai Ding, ^ab^ Hua Zhai, ^a*^ Hao Gong, ^bc^ Xiao Long, ^ab^ Yuanzhuo Tian, ^ab^ Zhanyong Hong, ^bc^ Long Zheng, ^bcd^ Xucong Wang, ^bc^ Xiangyu Chen, ^bc*^ Zhong Lin Wang, ^bc*^

a Anhui Province Key Lab of Aerospace Structural Parts Forming Technology and Equipment, Institute of Industry & Equipment Technology, Hefei University of Technology, Hefei, China.

b Beijing Key Laboratory of High-Entropy Energy Materials and Devices, Beijing Institute of Nanoenergy and Nanosystems, Chinese Academy of Sciences, Beijing, China

c School of Nanoscience and Engineering, University of Chinese Academy of Sciences, Beijing, China

d College of Polymer Science and Engineering, State Key Laboratory of Polymer Materials Engineering, Sichuan University, Chengdu, China

* Correspondence: [jxzhaihuajx@hfut.edu.cn](mailto:jxzhaihuajx@hfut.edu.cn) (H.Z.), [chenxiangyu@binn.cas.cn](mailto:chenxiangyu@binn.cas.cn) (X.Y.C.) [zhong.wang@mse.gatech.edu](mailto:zhong.wang@mse.gatech.edu) (Z.L.W.)

**Figure S1.** The transmission part and four modules of the HP-HNG.

**Figure S2.** Physical image of the HP-HNG.

**Figure S3.** Schematic diagram of the transmission part.

***Heave: a.*** *When the input shaft 15 rotational speed is less than or equal to the driven small gear 8/9 speed, the one-way bearing is in engaged operation, and the reciprocating motion of the buoy device is transmitted through the one-way bearing to shaft 15-gear 6 meshes with gear 4-large sun gear 3-planet carrier H.*
***b.*** *When the input shaft 15 rotational speed is greater than the driven small gear 8/9 speed, the system separates, and HP-HNG is decoupled from the gear-rack transmission system.*

***Pitch: a.*** *When the rotational speed of shaft 14 is less than or equal to the rotational speed of gear 11/gear 13, the one-way bearing is engaged, and the rotational motion of the swinging ball is transmitted through the one-way bearing to shaft 14-bevel gear 7 meshes with bevel gear 5-small sun gear 11-planet carrier H.*

***b.*** *When the rotational speed of shaft 14 is greater than the rotational speed of gear 11/gear 13, the one-way bearing is disengaged. Therefore, the swinging ball's motion and the HP-HNG side equations are calculated separately. At this time, the one-way bearing inputs a torque* $T_{c}=0$ *to shaft 18.*

**Figure** **S4.** Comparison of peak power between the HP-HNG and other hybrid nanogenerators.[1–13]

**Figure S5.** (a) Physical diagram of the circuit board. (b) The electrode of the M-TENG and the inductive electrode.

**Figure S6.** (a)The relationship between resistance $R_{1}$ on the circuit board and the output current (8x voltage). (b) The relationship between resistance $R_{2}$ on the circuit board and the output current (8x voltage).

**Figure S7.** Output current of the EMG pump at different rotational speeds.

**Figure S8.** Output voltage of each circuit board.

**Figure S9.** The $Q_{sc}$ and $V_{oc}$,of the M-TENG.

**Figure S10.** The relationship between the $V_{oc}$, $I_{sc}$, and load resistance of the M-TENG.

**Figure** **S11.** Diagram of the R-TENG's electrodes and friction layer.

**Figure S12.** Top view of the R-EMG coil connection.

**Figure S13.** The relationship between the R-EMG (a) $I_{sc}$, (b) $V_{oc}$, and rotational speed.

**Figure S14.** Peak power curves of the R-EMG at different rotation speeds.

**Figure S15.** The number of the R-EMG in series and the relationship between the (a) $V_{oc}$ and (b) $I_{sc}$ of the R-EMG.

**Figure S16.** Voltage generated by different positive materials in the R-TENG when rubbed with PTFE.

**Figure S17.** The relationship between the (a) $I_{sc}$, (b) $V_{oc}$, and (c) $Q_{sc}$ of the R-TENG and the rotational speed.

**Figure S18.** The relationship between the (a) $I_{sc}$, (b) $V_{oc}$, and (c) $Q_{sc}$ of the R-TENG and the number of the R-TENGs connected in parallel.

**Figure S19.** SEM images of the PTFE film surface in the R-TENG1 at (a) the initial state and (b) after 100 hours of working.

**Figure S20.** Maximum average power of M-TENG, R-TENG, and R-EMG

**Figure S21.** (a) Pendulum ball model. (b) Schematic Diagram of the HP-HNG heave and pitch.

**Figure S22.** The relationship of $I_{sc}$, $V_{oc}$, and $Q_{sc}$ of the M-TENG in (a)heave, (b)pitch, and (c)heave + pitch at a water wave frequency of 1 Hz.

**Figure S23.** Current variation curves of the EMG pump during heave, pitch, and combined heave + pitch motions of the HP-HNG.

**Figure S24.** Radar chart of the R-TENG's current with response to excitation waves generated at an interval of ${30}^{^{\circ}}$ from $0^{^{\circ}}$ to ${330}^{^{\circ}}$ .

**Figure S25.** (a) Relationship between the number of the R-TENGs and their current output when the HP-HNG undergoes pitch motion. (b) Relationship between the number of the R-TENGs and their charge output when the HP-HNG undergoes pitch motion. (c) Comparison of the R-TENG charge output during heave, pitch, and combined heave + pitch motions of the HP-HNG.

**Figure S26.** (a) When the water wave frequency is 1Hz, the HP-HNG undergoes heave, the relationship between the number of R-TENGs in parallel and the R-TENG voltage. Amplified voltage diagram of the R-TENG during heaving. (b) When the water wave frequency is 1Hz, the HP-HNG undergoes pitch, the relationship between the number of the R-TENGs in parallel and the R-TENG voltage. Amplified voltage diagram of the R-TENG during pitching. (c) When the water wave frequency is 1Hz, the HP-HNG undergoes heave, pitch, and combined heave + pitch motions, the voltage variation diagram for a single R-TENG.

**Figure S27** (a) The current variation of a single R-EMG when the water wave frequency is 1 Hz and the HP-HNG undergoes pitch motion. Amplified current diagram of R-EMG during pitching. (b) The current variation of a single R-EMG when the water wave frequency is 1 Hz and HP-HNG undergoes heave+pitch motions. Amplified current diagram of R-EMG during heave+pitch motions.

**Figure S28** (a) Voltage variation of a single R-EMG when the water wave frequency is 1 Hz and the HP-HNG undergoes heave motion. Amplified voltage diagram of R-EMG during heaving. (b) Voltage variation of a single R-EMG when the water wave frequency is 1 Hz and the HP-HNG undergoes pitch motion. Amplified voltage diagram of R-EMG during pitching. (c) Voltage variation of a single R-EMG when the water wave frequency is 1 Hz and the HP-HNG undergoes heave, pitch, and combined heave+pitch motions.

Figure **S29.** Circuit diagram of the HP-HNG.

**Figure S30.** Circuit diagram of the PMC.

*R-TENG1-8, M-TENG, and R-EMG1-4 in the circuit diagram represent the symbols after rectification.*

**Figure S31.** Selection of the (a) GDT and (b) $C_{0}$ for the management circuit.

**Figure S32.** Stored charge on the 1 mF capacitor charged by the R-TENG with and without the PMC.

**Figure S33.** (a) Charging curves of a single module powering different capacitors $C_{1}$ at a motor speed of 250 rpm in a lab setting. (b) Voltage curves on a 2.2 mF capacitor charged by a module with the PMC for different rotational speeds.

**Figure S34.** Charging curves for different sections within a single module when the device undergoes (a) heave or (b) pitch in a water wave environment.

**Figure S35.** Energy charging curves of the HP-HNG powering a capacitor in a water wave environment at 1 Hz.

**Figure S36.** The relationship between water wave frequency and energy generated by the HP-HNG

**Note S1 The motion process of the HP-HNG and the corresponding dynamic model.**

When capturing wave energy, waves cause ocean buoys to undergo pendulum and pitching motions, converting wave energy into mechanical energy.

When viewed from above, as pendulum ball a rotates counterclockwise, both internal gear 12 and external gear 10 rotate counterclockwise. However, due to the action of one-way bearing α, internal gear 12 meshes with gear 13 internally. Gear 13 operates counterclockwise, causing drive shaft 14 to rotate counterclockwise. When pendulum ball a rotates clockwise, both internal gear 12 and external gear 10 rotate clockwise. However, due to the action of one-way bearing β, external gear 9 meshes externally with gear 10, causing only gear 10 to operate counterclockwise. Drive shaft 14 continues rotating counterclockwise. Bevel gear 7 meshes with bevel gear 5, thereby driving sun gear 1 to rotate.

From the front view perspective, when the float moves upward relative to support column 16, the gear moves upward relative to the rack. Under the action of one-way bearing 8, only gear 1 operates, rotating counterclockwise, causing drive shaft 5 to rotate counterclockwise; When the float moves downward relative to support column 16, the gear moves downward relative to the rack. Under the action of one-way bearing 15, only gear 14 operates, rotating counterclockwise, and drive shaft 5 continues to rotate counterclockwise. Gear 2 meshes with gear 3, thereby driving large sun gear 4 to rotate. Through the combined action of the large sun gear 4 and the small sun gear 13, the planetary gear 6 achieves rotational speed increase. Consequently, the planetary carrier 16 rotates faster, imparting greater rotational speed to the connected generator, thereby enhancing power generation efficiency.

**Note S2 Calculation of the rotational speed of the power generation modules.**

Dynamic Analysis of Gear Transmission Mechanisms

Input-Output Transmission Ratio

$$n_{7}=-\frac{z_{5}}{z_{7}}n_{5}$$

$$n_{5}=n_{1}$$

$$n_{6}=-\frac{z_{4}}{z_{6}}n_{4}$$

$$n_{4}=n_{3}$$

$$v_{rack}=\frac{n_{8}\pi mz_{8}}{60}$$

$$n_{8}=n_{6}$$

$$n_{13}=\frac{z_{12}}{z_{13}}n_{12}$$

$$n_{13}=n_{7}$$

$$n_{12}=n_{a}$$

$$i_{1-3}^{H}=\frac{n_{1}-n_{H}}{n_{3}-n_{H}}=-\frac{z_{3}}{z_{1}}$$

$$n_{H}=2n_{a}+\frac{12v_{rack}}{\pi}$$

$n_{H}$ and $n_{HP-HNG}$ are synchronous belt drives with a transmission ratio of 1/2.

$$n_{HP-HNG}=4n_{a}+\frac{{24v}_{rack}}{\pi}$$

| ***Gear Number*** | $\boldsymbol{z}_{\boldsymbol{1}}$ | $\boldsymbol{z}_{\boldsymbol{2}}$ | $\boldsymbol{z}_{\boldsymbol{3}}$ | $\boldsymbol{z}_{\boldsymbol{4}}$ | $\boldsymbol{z}_{\boldsymbol{5}}$ | $\boldsymbol{z}_{\boldsymbol{6}}$ | $\boldsymbol{z}_{\boldsymbol{7}}$ | $\boldsymbol{z}_{\boldsymbol{8}}$ | $\boldsymbol{z}_{\boldsymbol{9}}$ | $\boldsymbol{z}_{\boldsymbol{10}}$ | $\boldsymbol{z}_{\boldsymbol{11}}$ | $\boldsymbol{z}_{\boldsymbol{12}}$ | $\boldsymbol{z}_{\boldsymbol{13}}$ |
| --- | --- | --- | --- | --- | --- | --- | --- | --- | --- | --- | --- | --- | --- |
| ***Rotational speed*** | $\boldsymbol{n}_{\boldsymbol{1}}$ | $\boldsymbol{n}_{\boldsymbol{2}}$ | $\boldsymbol{n}_{\boldsymbol{3}}$ | $\boldsymbol{n}_{\boldsymbol{4}}$ | $\boldsymbol{n}_{\boldsymbol{5}}$ | $\boldsymbol{n}_{\boldsymbol{6}}$ | $\boldsymbol{n}_{\boldsymbol{7}}$ | $\boldsymbol{n}_{\boldsymbol{8}}$ | $\boldsymbol{n}_{\boldsymbol{9}}$ | $\boldsymbol{n}_{\boldsymbol{10}}$ | $\boldsymbol{n}_{\boldsymbol{11}}$ | $\boldsymbol{n}_{\boldsymbol{12}}$ | $\boldsymbol{n}_{\boldsymbol{13}}$ |
| ***Number of teeth*** | ***12*** | ***18*** | ***48*** | ***20*** | ***15*** | ***40*** | ***30*** | ***20*** | ***20*** | ***48*** | ***20*** | ***100*** | ***20*** |
| ***Module*** | ***2*** | ***2*** | ***2*** | ***2*** | ***2*** | ***2*** | ***2*** | ***2*** | ***2*** | ***2*** | ***2*** | ***2*** | ***2*** |

$v_{rack}$ *Rack speed (mm/s)*

$n_{H}$ *Planetary carrier speed*

$n_{a}$ *Pendulum ball speed*

$n_{HP-HNG}$*Power generation module*

**Note S3 Derivation process of the heave-pitch equation**

Dynamic Model of a Pendulum

Consider a ground-based inertial coordinate system OXYZ, where the Z-axis points vertically upward. The buoy undergoes a heave motion about the Y-axis with an angle of heave $\varphi$. A pendulum is suspended above the buoy’s bulkhead at a vertical distance h; its length is l, and the angle of oscillation of the pendulum ball relative to the buoy is $\theta$ (assuming the oscillation occurs within the buoy’s longitudinal plane).

Suppose the buoy rotates about a fixed point O. The coordinates of the pivot point P in the buoy’s coordinate system are (0, 0, h). The position of the pendulum ball relative to the pivot point in the buoy’s coordinate system is ($l sin \theta$, 0, $-l cos \theta$). By applying a rotation transformation (rotation about the Y-axis by $\varphi$), we obtain the coordinates of the pendulum ball in the ground-based coordinate system:

$$X=l\sin\left( \theta+\varphi\right)-h\sin\varphi$$

$$Y=0$$

$$Z=h\cos\varphi-l\cos\left( \theta+\varphi\right)$$

Taking the derivative with respect to time yields the velocity components in the X, Y, and Z directions:

$$\dot{X}=l\left( \dot{\theta+\dot{\varphi}} \right)\cos\left( \theta+\varphi\right)-h\dot{\varphi}\cos\varphi$$

$$\dot{Z}= l\left( \dot{\theta+\dot{\varphi}} \right)\sin\left( \theta+\varphi\right)-h\dot{\varphi}\sin\varphi$$

After expanding the squares of $\dot{Ẋ}$ and$\dot{Ż}$ and adding them:

$$\dot{X}^{2}+\dot{Z}^{2}=l^{2}\left( \dot{\theta}+\dot{\varphi} \right)^{2}+h^{2}\dot{\varphi}^{2}-2lh\dot{\varphi}\left( \dot{\theta}+\dot{\varphi} \right)\cos\theta$$

$$=l^{2}\dot{\theta}^{2}+\dot{\varphi}^{2}\left( l^{2}+h^{2}-2lh\cos\theta\right)+2l\dot{\theta}\dot{\varphi}\left( l-h\cos\theta\right)$$

The kinetic energy of the swinging ball is:

$$T=\frac{1}{2}m_{1}\left[ \left( l\dot{\theta}\sin\theta+h\dot{\varphi} \right)^{2}+\left( l\dot{\varphi}\cos\theta\right)^{2}+\left( l\dot{\theta}\cos\theta\right)^{2} \right]$$

The potential energy of the pendulum is:

$$V=m_{1}g\left[ h\cos\theta-l\cos\left( \theta+\varphi\right) \right]$$

Derive the heaving and pitching motion equations using the Lagrange equation

The Lagrangian function is:

$$L=T-V$$

Let the torque acting on the pendulum bob (including added inertia force and damping) be:

$$f_{1}=-a\ddot{\theta}-b\dot{\theta}$$

According to the generalized force:

$$Q_{\theta}=2f_{1}$$

$$Q_{\theta}=-2a\ddot{\theta}-2b\dot{\theta}$$

For the generalized coordinate $\theta$, we get:

Pitching Equation

$$\frac{d}{dt}\left( \frac{\partial L}{\partial\dot{\theta}} \right)-\frac{\partial L}{\partial\theta}=Q_{\theta}$$

$$\frac{\partial L}{\partial\theta}=\frac{\partial T}{\partial\theta}-\frac{\partial V}{\partial\theta}$$

Calculate partial derivatives:

$$\frac{\partial L}{\partial\dot{\theta}}=m_{1}\left( l^{2}\dot{\theta}+lh\dot{\varphi}\sin\theta\right)$$

$$\frac{d}{dt}\left( \frac{\partial L}{\partial\dot{\theta}} \right)=m_{1}\left[ l^{2}\ddot{\theta}+lh\left( \ddot{\varphi}\sin\theta+\dot{\varphi}\dot{\theta}\cos\theta\right) \right]$$

$$\frac{\partial T}{\partial\theta}=m_{1}\left( -l^{2}\dot{\varphi}^{2}\cos\theta\sin\theta+lh\dot{\theta}\dot{\varphi}\cos\theta\right)$$

$$\frac{\partial V}{\partial\theta}=m_{1}gl\sin\left( \theta+\varphi\right)$$

$$\frac{\partial L}{\partial\theta}=m_{1}\left( -l^{2}\dot{\varphi}^{2}\cos\theta\sin\theta+lh\dot{\theta}\dot{\varphi}\cos\theta-gl\sin\left( \theta+\varphi\right) \right)$$

Substitute into Lagrange equation to get:

$$\ddot{\theta}+\frac{2b}{m_{1}l^{2}+2a}\theta+\frac{m_{1}l}{m_{1}l^{2}+2a}\left( h\ddot{\varphi}+\dot{\varphi}^{2}\cos\theta+g\sin\varphi\right)\sin\theta=0$$

$\varphi$ *Overall tilt angle of the device*

$\theta$ *Angle of oscillation of the pendulum ball around its central axis*

$l$ *Radius of rotation*

$m₁$ *Mass of the pendulum ball*

$a,b$*Viscous mass coefficient, Viscous damping coefficient*

$h$ *Distance from the pendulum ball to the plate*

$f₁$*Resistance of the single-sided longitudinal oscillation transmission device*

Establishment of the heave-pitch Coupled Equation

Heave Dynamics Equation

$$m\ddot{x}=F\left( t,x,\dot{x},\ddot{x} \right)-F_{1}$$

$x$ *Displacement in the buoy heave direction*

$m$ *Total mass of the buoy*

$F(t,x,ẋ,ẍ)$ *Hydrodynamic force exerted by waves*

$F₁$ *Force driving transmission among components within the buoy*

$$F\left( t,x,\dot{x},\ddot{x} \right)=F_{a}\left( t \right)+F_{b}\left( t \right)+F_{c}\left( \dot{x,}\ddot{x} \right)$$

$F_{a}$ *Wave excitation force applied by the incident wave*

$F_{b}$ *Hydrostatic pressure due to vertical oscillation*

$$F_{b}=k_{a}x=\rho g\pi R^{2}x$$

$$k_{a}=\rho g\pi R^{2}$$

$F_{c}\left( \dot{x},\ddot{x} \right)$ *represents the hydrodynamic forces acting on the buoy due to its own pendulum motion in the absence of incident waves.*

$$F_{c}\left( \dot{x},\ddot{x} \right)=-m_{2}\ddot{x}-c_{1}\dot{x}$$

$m_{2}$ *Added mass*

$c_{1}$ *Added damping, Heave coefficient*

$m_{a}$ *Equivalent damping*

$c_{a}$ *Equivalent mass*

Parameters to consider in pitch motion

$$\bar{GM}=\bar{KM}-\bar{KG}$$

$$\bar{BM}=\frac{I_{1}}{V}$$

$\bar{GM}$ *Initial stability height*

$\bar{KM}$ *Distance from initial stability center to baseline*

$\bar{KG}$ *Center of gravity height*

$\bar{BM}$ *Initial stability radius*

$I₁$ *Waterline surface moment of inertia*

$V$ *Displacement volume*

Restoration stiffness $\rho gV\bar{GM}$ is a function of the platform's displacement volume V and initial stability height $\bar{GM}$. Since $\bar{KG}$ remains constant, $\bar{BM}$ equals the ratio of the waterline surface moment of inertia to the displacement volume, whose value changes minimally. The primary factor influencing$\bar{GM}$ is $\bar{KM}$. When considering only the pendulum motion of ocean buoys and wave surface elevation, the expressions for $\bar{GM}$ and $V$ are:

$$\bar{GM}=\bar{{GM}_{0}}-\frac{1}{2}\left[ x\left( t \right)-\xi\left( x_{1},y_{1},t \right) \right]$$

$$V=V_{0}-A_{1}\left[ x\left( t \right)-\xi\left( x_{1},y_{1},t \right) \right]$$

$A₁$ *Surface area of the waterline*

$\xi(x₁,y₁,t)$ *Position of the buoyant center at wave elevation*

The initial stability height is the primary indicator for measuring a ship's initial stability. Typically, at small angles of heel, the greater the initial stability height, the larger the restoring moment, and the stronger the ship's ability to resist heeling moments. The formula for calculating initial stability height is $GM = KM - KG$, where $KM$ is the distance from the baseline to the initial transverse metacenter, and $KG$ is the height of the ship's center of gravity. A higher initial stability height indicates superior stability for the vessel. Therefore, selecting an appropriate initial stability height is crucial during ship design.

$k_{a}$ *Longitudinal Pitch Recovery Stiffness*

$$k_{a}=\rho gV\bar{GM}=\rho g\left\{ \bar{{GM}_{0}}-\frac{1}{2}\left[ x\left( t \right)-\xi\left( x_{1},y_{1},t \right) \right] \right\}\left\{ V_{0}-A_{1}\left[ x\left( t \right)-\xi\left( x_{1},y_{1},t \right) \right] \right\}$$

$$k_{a}=\rho g\bar{{GM}_{0}}V_{0}-\frac{1}{2}\rho g\left( V_{0}+2A_{1}\bar{{GM}_{0}} \right)\left[ x\left( t \right)-\xi\left( x_{1},y_{1},t \right) \right]+\frac{1}{2}\rho gA_{1}\left[ x\left( t \right)-\xi\left( x_{1},y_{1},t \right) \right]^{2}$$

Considering only the influence of ocean buoy heave motion on pitch motion, further reasonable simplifications yield the following equations for heave and pitch motion:

Heave Equation

$$\left( m+m_{a}+m_{2} \right)\ddot{x}\left( t \right)+\left( c_{1}+c_{a} \right)\dot{x}\left( t \right)+\rho gA_{1}x\left( t \right)=F_{1}\cos\left( \omega t \right)$$

Pitch Equation

$$\left( I_{2}+A_{2} \right)\ddot{\varphi}\left( t \right)+c_{2}\dot{\varphi}\left( t \right)+\rho gV\bar{GM}\varphi\left( t \right)=M_{1}\cos\left( \omega t \right)$$

$I₂$ *Buoy roll moment of inertia* $A₂$*Additional roll moment of inertia* $c₂$*Roll damping coefficient* $M₁$ *Roll wave moment amplitude*

$$\left( I_{2}+A_{2} \right)\ddot{\varphi}\left( t \right)+c_{2}\dot{\varphi}\left( t \right)+\rho gV_{0}\left\{ \bar{{GM}_{0}}-\frac{1}{2}\left[ x\left( t \right)-\xi\left( x_{1},y_{1},t \right) \right] \right\}\varphi\left( t \right)=M_{1}\cos\left( \omega t \right)$$

When considering the coupled motion of heave and roll in ocean buoys,

Pitch Equation

$$\left( I_{2}+A_{2} \right)\ddot{\varphi}\left( t \right)+c_{2}\dot{\varphi}\left( t \right)+\rho gV_{0} \varphi\left( t \right)-k_{3}\varphi\left( t \right)x\left( t \right)=M_{1}\cos\left( \omega t \right)$$

$k₃ = \rho gh₂A₁$*Nonlinear coefficient: Nonlinear restoring torque per unit heave displacement causing unit pitch motion*

$h₂$ *Vertical distance from the hydrostatic surface to the buoy center of gravity.*

$F_{1}$ *Heave Wave Excitation Amplitude*

Coupled Equation of Buoy heave and pitch

$$\left( m+m_{a}+m_{2} \right)\ddot{x}+\left( c_{1}+c_{a} \right)\dot{x}+\left( k_{a}+k_{1}+k_{2} \right)x=F_{1}\cos\left( \omega t \right)$$

$$\left( I_{2}+A_{2} \right)\ddot{\varphi}\left( t \right)+c_{2}\dot{\varphi}\left( t \right)+\rho gV_{0} \varphi\left( t \right)-k_{3}\varphi\left( t \right)x\left( t \right)=M_{1}\cos\left( \omega t \right)$$

$$\ddot{\theta}+\frac{2b}{m_{1}l^{2}+2a}\theta+\frac{m_{1}l}{m_{1}l^{2}+2a}\left( h\ddot{\varphi}+\dot{\varphi}^{2}\cos\theta+g\sin\varphi\right)\sin\theta=0$$

**Table S1** Comparison of power density and test conditions of the HP-HNG with different devices

| Device structure | Power Density | Test Conditions | Refs |
| --- | --- | --- | --- |
| FH-HG | 4.76 | Water wave 1Hz | [40] |
| TEP-HEH | 5.73 | Water wave 0.5Hz | [41] |
| M-TEHG | 6.041 | Water wave 0.75Hz | [42] |
| iTEHG | 7.25 | Water wave 1.47Hz | [43] |
| TEHG | 10.1 | Water wave 1Hz | [44] |
| FH-HG | 18.98 | Water wave 1.4Hz | [45] |
| MHTEG | 20.9 | Water wave 1.3Hz | [46] |
| HTEG | 24.1 | Water wave 0.5Hz | [47] |
| MBC-TENG+RD-EMG | 30.7 | Water wave 1.05Hz | [48] |
| SSTE-HNG | 31.6 | Water wave 0.7Hz | [49] |
| TENG+EMG | 39.59 | Water wave 2.4Hz | [50] |
| EM-TENG | 50.053 | Water wave 1Hz | [51] |
| SPC-HEH | 82.4 | Water wave 2Hz | [52] |
| **This work** | **127.303** | **Water wave 1Hz** | **-** |

**Supporting Movies**

Movie **S1** The HP-HNG self-powered thermometer at a water wave frequency of 1 Hz.

Movie **S2** The HP-HNG self-powered buzzer at a water wave frequency of 1 Hz.

Movie **S3** The HP-HNG drives the optoelectronic sensor at a water wave frequency of 1 Hz.

Movie **S4** When the water wave frequency is 1 Hz, the HP-HNG drives a small motor.

**Reference**

1 Y. Hu, H. Qiu, Q. Sun, Z.L. Wang, L. Xu, “Wheel‐Structured Triboelectric Nanogenerators with Hyperelastic Networking for High‐Performance Wave Energy Harvesting,” *Small Methods* 7 (2023):2300582. <https://doi.org/10.1002/smtd.202300582>.

2 W. Gao, X. Gao, J. Su, et al., “Gyro‐Multigrid Triboelectric Nanogenerator via Topological Defects Strategy for Efficiently Harvesting Low‐Grade Wave Energy,” *Advanced Energy Materials* (2025):2405398. <https://doi.org/10.1002/aenm.202405398>.

3 S. Tian, X. Wei, L. Lai, et al., “Frequency Modulated Hybrid Nanogenerator for Efficient Water Wave Energy Harvesting,” *Nano Energy* 102 (2022):107669. <https://doi.org/10.1016/j.nanoen.2022.107669>.

4 Q. Xu, C. Shang, H. Ma, et al., “A Guided-Liquid-Based Hybrid Triboelectric Nanogenerator for Omnidirectional and High-Performance Ocean Wave Energy Harvesting,” *Nano Energy* 109 (2023):108240. <https://doi.org/10.1016/j.nanoen.2023.108240>.

5 C. Zhang, W. Yuan, B. Zhang, et al., “High Space Efficiency Hybrid Nanogenerators for Effective Water Wave Energy Harvesting,” *Advanced Functional Materials* 32 (2022):2111775. <https://doi.org/10.1002/adfm.202111775>.

6 C. Chen, D. Guo, L. Tuo, et al., “One Meter Triboelectric Nanogenerator for Efficient Harvesting of Meter‐Scale Wave Energy,” *Advanced Functional Materials* 34 (2024):2406775. <https://doi.org/10.1002/adfm.202406775>.

7 Y. Yu, X. Cheng, H. Li, Z.L. Wang, T. Cheng, “Power Improvement of Triboelectric Nanogenerator by Morphological Transformation Strategy for Harvesting Irregular Wave Energy,” *Chemical Engineering Journal* 490 (2024):151897. <https://doi.org/10.1016/j.cej.2024.151897>.

8 H. Jung, Z. Lu, W. Hwang, et al., “Modeling and Sea Trial of a Self-Powered Ocean Buoy Harvesting Arctic Ocean Wave Energy Using a Double-Side Cylindrical Triboelectric Nanogenerator,” *Nano Energy* 135 (2025):110641. <https://doi.org/10.1016/j.nanoen.2024.110641>.

9 W. Zhong, L. Xu, X. Yang, et al., “Open-Book-Like Triboelectric Nanogenerators Based on Low-Frequency Roll-Swing Oscillators for Wave Energy Harvesting,” *Nanoscale* 11 (2019):7199–7208. <https://doi.org/10.1039/C8NR09978B>.

10 J. Zhang, Y. Yu, H. Li, et al., “Triboelectric-Electromagnetic Hybrid Generator with Savonius Flapping Wing for Low-Velocity Water Flow Energy Harvesting,” *Applied Energy* 357 (2024):122512. <https://doi.org/10.1016/j.apenergy.2023.122512>.

11 B. Yang, H. Li, Z. Wang, et al., “High-Performance Triboelectric Nanogenerator Inspired by Bionic Jellyfish for Wave Energy Harvesting,” *Chemical Engineering Journal* 503 (2025):158399. <https://doi.org/10.1016/j.cej.2024.158399>.

12 B. Zhao, Z. Li, X. Liao, et al., “A Heaving Point Absorber-Based Ocean Wave Energy Convertor Hybridizing a Multilayered Soft-Brush Cylindrical Triboelectric Generator and an Electromagnetic Generator,” *Nano Energy* 89 (2021):106381. <https://doi.org/10.1016/j.nanoen.2021.106381>.

13 C. Zhang, Y. Hao, X. Lu, et al., “Passive Wireless Marine Internet-of-Things Buoy Based on Hybrid Nanogenerator with Omnidirectional Water-Wave Energy Harvesting,” *Nano Energy* 138 (2025):110809. <https://doi.org/10.1016/j.nanoen.2025.110809>.
